# Endogenous Opioid Levels Do Not Correlate With Itch Intensity and Therapeutic Interventions in Hepatic Pruritus

**DOI:** 10.3389/fmed.2021.641163

**Published:** 2021-04-14

**Authors:** Miriam M. Düll, Katharina Wolf, Marcel Vetter, Peter Dietrich, Markus F. Neurath, Andreas E. Kremer

**Affiliations:** ^1^Department of Medicine 1, University Hospital Erlangen and Translational Research Center, Friedrich-Alexander-University Erlangen-Nürnberg, Erlangen, Germany; ^2^Emil-Fischer-Zentrum, Institute of Biochemistry, Friedrich-Alexander-University Erlangen-Nürnberg, Erlangen, Germany; ^3^Deutsches Zentrum Immuntherapie DZI, Erlangen, Germany

**Keywords:** endorphin, dynorphin, enkephalin, rifampicin, bezafibrate, cholestasis, liver, therapy

## Abstract

**Background:** Chronic pruritus affects up to 70% of patients with immune-mediated hepatobiliary disorders. Antagonists of the μ-opioid receptor (MOR) and agonists of the κ-opioid receptor (KOR) are used to treat hepatic itch, albeit with limited success. An imbalance between ligands of MOR and KOR receptors has recently been suggested as a potential mechanism of hepatic pruritus. In this study, we therefore investigated systemic levels of important endogenous opioids such as β-endorphin, dynorphin A, Leu- and Met-enkephalin in plasma of a large cohort of well-characterized patients with immune-mediated cholestatic disorders, including patients with liver cirrhosis, and during effective anti-pruritic therapy.

**Methods:** Plasma samples and clinical data were prospectively collected from well-characterized patients with primary/secondary sclerosing cholangitis (PSC/SSC), primary biliary cholangitis (PBC) and overlap syndromes suffering from pruritus (*n* = 29) and age-, gender- and disease-matched controls without pruritus (*n* = 27) as well as healthy controls (*n* = 20). General laboratory testing for hepatobiliary and renal function was performed. Levels of β-endorphin, dynorphin A, Leu- and Met-enkephalin were quantified in plasma by ELISA. Intensity of pruritus over the last week was evaluated using a visual analog scale (VAS, 0–10).

**Results:** PBC and PSC patients with or without pruritus did neither differ in disease entity, disease stage, nor in the presence of cirrhosis. While both dynorphin A and β-endorphin concentrations were lower in pruritic patients compared to those without pruritus and healthy controls, the MOR/KOR ligand ratio was unaltered. No significant differences were observed for Leu- and Met-enkephalin concentrations. Opioid levels correlated with neither itch intensity nor stage of disease. Cirrhotic patients displayed higher concentrations of MOR agonist Leu-enkephalin and KOR agonist dynorphin A. Endogenous opioid levels remained largely unchanged after successful treatment with the potent anti-pruritic drugs rifampicin and bezafibrate.

**Conclusions:** Endogenous opioid levels and the MOR/KOR ligand ratio neither correlate with itch intensity nor differentiate pruritic from non-pruritic patients with immune-mediated liver diseases. Thus, endogenous opioids may modulate signaling pathways involved in hepatic pruritus, but are unlikely to represent the major pruritogens in liver disease.

## Introduction

Chronic pruritus, defined as itch lasting for 6 weeks or more, represents a serious and challenging symptom in several systemic diseases, including chronic kidney disease (CKD), hematological disorders, and hepatobiliary diseases (1, 2). Up to one fifth of patients with generalized pruritus suffers from a systemic disease (3). Hepatobiliary disorders are associated with various extrahepatic manifestations such as abdominal pain, jaundice, fatigue, but also pruritus.

Itch is more frequently prevalent in cholestatic liver diseases, which can result from reduced bile secretion and/or flow on the level of hepato- and/or cholangiocytes (4).

Chronic immune-mediated cholestatic liver diseases such as primary biliary cholangitis (PBC) and primary sclerosing cholangitis (PSC) are commonly associated with pruritus. Up to 70% of patients are affected during their course of disease (4). A significant proportion of these patients suffers from moderate to severe pruritus, which can dramatically diminish quality of life and in extreme cases, even evoke suicidal intentions (5).

Several substances, including bile salts, endogenous opioids, histamine, serotonin, progesterone metabolites and lysophosphatidic acid have been suggested as potential pruritogens. Still, the causal receptors and signaling pathways remain elusive (6).

The endogenous opioid system represents one potential factor in hepatic pruritus (7). This hypothesis is supported by the fact that itch is commonly reported as adverse effect by patients receiving opioids, in particular if administered spinally (8). Concentrations of endogenous opioids were elevated in rats after surgically-induced cholestasis (9, 10) and in a few cholestatic PBC patients (11, 12). The mRNA expression of the opioid precursor molecule preproenkephalin was found in liver tissue of cholestatic rats (13). Increased Met-enkephalin immunoreactivity was observed in liver tissue of patients with PBC or chronic hepatitis C virus infection (14, 15). Antagonists of the μ-opioid receptor (MOR) such as naloxone or naltrexone and agonists of the κ-opioid receptor (KOR) such as nalfurafine are used to treat hepatic itch, albeit with limited anti-pruritic effect (16, 17). Amongst other findings, this data led to the hypothesis that substances activating μ-opioid receptors (MOR) might induce itch while agonists of κ-opioid receptor (KOR) rather activate anti-pruritogenic pathways.

However, no correlation between systemic endogenous opioid levels and intensity of pruritus could be established so far (18).

A recent study explained this lack of correlation with the finding of an imbalance in human plasma between ligands of μ-opioid receptors (β-endorphin) and κ-opioid receptors (dynorphin A) in patients with liver diseases and pruritus. It was concluded that a disproportion between MOR and KOR binding endogenous opioids (MOR/KOR ligand ratio) might contribute to hepatic pruritus (19).

In this study, we quantified levels of important endogenous opioids including β-endorphin, dynorphin A, Leu- and Met-enkephalin in plasma of a large cohort of well-characterized patients with immune-mediated cholestatic disorders. In addition, we analyzed the change of endogenous opioid levels during effective anti-pruritic therapy.

## Materials and Methods

### Patient Characteristics and Sampling

Plasma samples (in blood-sampling tubes containing ethylene-diamine-tetra-acetic acid, EDTA; Sarstedt, Nümbrecht, Germany) and clinical data were prospectively collected at the University Hospital Erlangen, Germany between 2014 to 2020. Plasma samples were centrifuged at 800 g for 10 min followed by immediate storage of plasma at −80°C until further testing.

The investigated cohort consisted of patients with primary or secondary sclerosing cholangitis (PSC/SSC), primary biliary cholangitis (PBC) and overlap syndromes. We included patients suffering from pruritus (*n* = 29) and age-, gender- and disease-matched controls without pruritus (*n* = 27) as well as age- and gender-matched healthy controls (*n* = 20). General laboratory testing for the hepatobiliary and renal function was performed, including assessing the values of AST (aspartate aminotransferase), ALT (alanine aminotransferase), AP (alkaline phosphatase), γGT (gamma-glutamyltransferase), total bilirubin, albumin, INR (International Normalized Ratio) and creatinine. None of the tested patients received an oral opioid antagonist (e.g., naloxone and naltrexone) or opioid-containing pain medication prior to blood sampling collection. Prevalence of cirrhosis was determined non-invasively by elastography or liver biopsy results. To evaluate the change in endogenous opioid levels during antipruritic treatment, we additionally included 22 patients who either received rifampicin (150–300 mg qd) or bezafibrate (400 mg qd) for 2–6 weeks. Except for the separately evaluated patient group treated with either rifampicin or bezafibrate, pruritic patients included in this study did not receive any oral or topical anti-pruritic drugs except for emollient or hydrating topical agents.

Clinical data and laboratory work-up of all patients are summarized in Tables 1, 2. Upon their visit or admission to the hospital, patients were asked to state mean pruritus intensity over the last week on a visual analog scale (VAS, 0–10) as part of a questionnaire. Patients handed in a written informed consent according to the Declaration of Helsinki and this study was approved by the local ethics committee (approval number 238_13B).

**Table 1:** Clinical and laboratory characteristics of pruritic and non-pruritic patients as well as healthy controls.

	**Pruritus**	**No pruritus**	**Controls**
Number of subjects	29	27	20
Male^a^	9	8	6
Female^a^	20	19	14
Age^b^	52 (42–59)	50 (34–60)	52 (44–58)
Disease entity^a^			
PBC	13	7	—
PSC/SSC	12	14	—
Overlap syndromes	4	6	—
Cirrhosis^a^	7	6	—
Laboratory values^b,c^			*p*-value
ALT (GPT, [<50 U/L])^d^	63 (37–93)	48 (25–76)	0.17
AST (GOT, [<50 U/L])^d^	62 (39–84)	41 (31–79)	0.11
γGT (<40 U/L)^e^	152 (63–286)	121 (28–224)	0.17
AP [40–130 U/L]^f^	282 (144–384)	204 (120–323)	0.17
Total bilirubin (<1.1 mg/dl)	1.4 (0.8–2.5)	0.9 (0.7–1.7)	0.19
Albumin (35–55 g/L)	38.1 (36.3–42.9)	41.6 (38.8–42.9)	0.17
INR (0.85–1.15)	0.95 (0.92–1.03)	1.00 (0.95–1.05)	0.12
Creatinine (0.67–1.17 mg/dl)	0.73 (0.64–0.83)	0.71 (0.63–0.82)	0.61

*Data were statistically analyzed by Mann-Whitney-U-test*.

a *Data represent the respective number of subjects*.

b *Data are shown as median (interquartile range)*.

c *Reference values are shown in square brackets*.

d *Reference values according to gender, male: <50 U/L, female: <35 U/L*.

e *Reference values according to gender, male: <60 U/L, female: <40 U/L*.

f *Reference values according to gender, male: 40–130 U/L, female: 35–105 U/L*.

*PBC, primary biliary cholangitis; PSC/SSC, primary/secondary sclerosing cholangitis; AST, aspartate aminotransferase; ALT, alanine aminotransferase, AP, alkaline phosphatase, γGT, gamma-glutamyltransferase; INR, international normalized ratio*.

**Table 2:** Laboratory parameters in 22 patients before and after receiving anti-pruritic treatment with either rifampicin (150–300 mg qd) or bezafibrate (400 mg qd).

	**Pre-treatment**	**Post-treatment**	***P*-value**
**Laboratory values**^a^			
ALT (GPT, [<50 U/L])^b,c^	62 (40–100)	52 (38–62)	0.48
AST (GOT, [<50 U/L])^c^	93 (61–139)	84 (34–116)	0.55
γGT (<40 U/L)^d^	133 (62–250)	81(52–250)	0.18
AP (40–130 U/L)^e^	196 (161–323)	165 (150–282)	0.07
Total bilirubin (<1.1 mg/dl)	4.4 (1.3–8.3)	3.8 (2.3–5.5)	0.08
Albumin (35–55 g/L)	40.3 (32.6–41.3)	40.7 (30.5–42.3)	0.89
INR (0.85–1.15)	0.98 (0.96–1.27)	1.00 (0.93–1.09)	0.34
Creatinine (0.67–1.17 mg/dl)	0.86 (0.80–0.91)	0.72 (0.64–0.87)	0.11

*Data were statistically analyzed by Wilcoxon-test*.

a *Data are shown as median (interquartile range)*.

b *Reference values are shown in square brackets*.

c *Reference values according to gender, male: <50 U/L, female: <35 U/L*.

d *Reference values according to gender, male: <60 U/L, female: <40 U/L*.

e *Reference values according to gender, male: 40–130 U/L, female: 35–105 U/L*.

*AST, aspartate aminotransferase; ALT, alanine aminotransferase, AP, alkaline phosphatase, γGT, gamma-glutamyltransferase; INR, international normalized ratio*.

### Enzyme-Linked Immunosorbent Assays

Levels of β-endorphin, dynorphin A, Leu- and Met-enkephalin in plasma samples were quantified using commercial fluorescent EIA and ELISA kits (β-endorphin, dynorphin A, Leu-Enkephalin: Phoenix Pharmaceuticals, Burlingham, USA; Met-Enkephalin: MyBioSource, San Diego, USA) according to the respective manufacturer's guidelines. All ELISA kits had <15% intra-assay and <15% inter-assay variation. The cross-reactivity for peptides in % for the ELISA kits was as following: β-endorphin kit: 100% for β-endorphin (human), 100% for Ac-β-endorphin (human); dynorphin A kit: 100% for dynorphin A (human, rat, mouse, porcine), <0.1% for dynorphin A (1–13, porcine), and 0% for dynorphin A (1–8, porcine); Leu-enkephalin kit: 100% for Leu-Enkephalin (human), 0% for Met-enkephalin, Met-Enkephalin-Arg-Gly-Leu, Leu-Enkephalin-Arg; Met-Enkephalin kit: 100% for Met-Enkephalin (human), 0% for Leu-enkephalin.

### Data and Statistical Analyses

Statistical analyses were conducted with STATISTICA 7.0 software (StatSoft Inc., Tulsa, USA). Normality was evaluated by Shapiro-Wilk W-test. Non-parametric Mann-Whitney-U-test or Wilcoxon test was applied if data were not normally distributed and data were given as median with 25 and 75% quartiles. In case of normal distribution, values were depicted as mean ± standard error of means (SEM) and analyzed by multi-way analysis of variance (ANOVA) with least significant difference (LSD) *post hoc* testing. Spearman rank was applied to calculate correlations. Graphs and figures were generated with Origin 2020®, Corel Draw X7®, and Microsoft Excel®.

## Results

Patients with immune-mediated cholestatic liver diseases with (*n* = 29) and without pruritus (*n* = 27) did neither differ in disease entity, nor disease severity including the presence of liver cirrhosis (Table 1). Seventy percent of patients reporting on pruritus were female, in accordance with prior data indicating that women with cholestatic conditions are more often affected by pruritus than male patients (13–15). Laboratory values revealed no significant differences in transaminases levels, cholestasis parameters, surrogate markers for liver function (albumin, INR) or serum creatinine levels between pruritic and non-pruritic patients (Table 1). The mean pruritus VAS score over the last week was 6.2 ± 0.5 in the pruritus group. We additionally evaluated endogenous opioid levels in 22 patients pre-/post anti-pruritic treatment with either rifampicin (150–300 mg qd) or bezafibrate (400 mg qd). Transaminases and cholestasis parameters did not significantly differ before and after treatment (Table 2).

### β-Endorphin and Dynorphin A Levels Are Lower in Pruritic Patients Without a Change to the MOR/KOR Ligand Ratio

**Figure 1:**
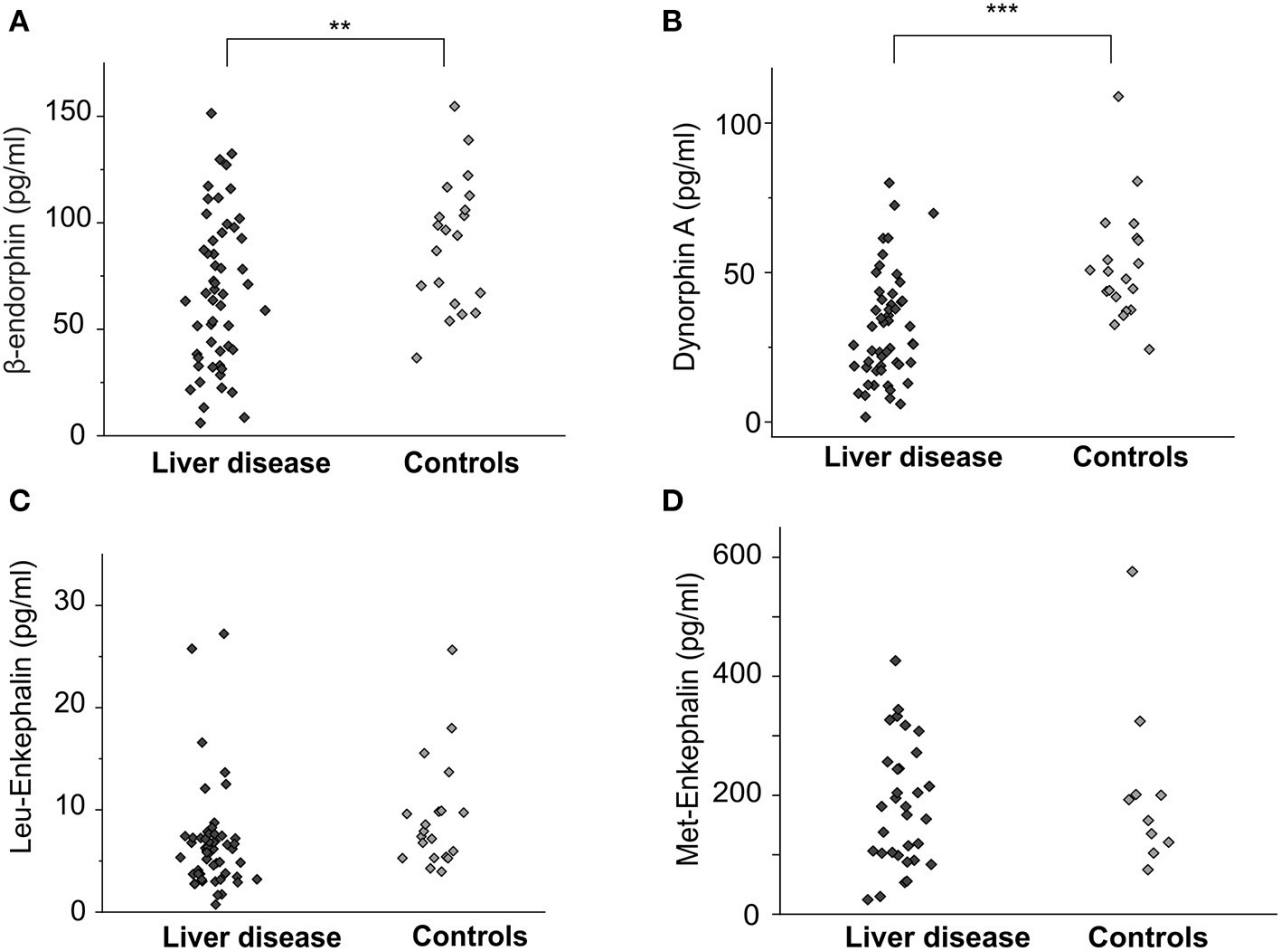
Endogenous opioid plasma levels in patients with cholestatic liver diseases and healthy controls. Concentrations [pg/ml] of β-endorphin, dynorphin A, Leu-enkephalin and Met-enkephalin of patients with cholestatic liver diseases (*n* = 56, dark gray diamonds) and age- and gender matched controls (*n* = 20, light gray diamonds) are shown as individual values in **(A–D)**. Significantly lower levels of both β-endorphin **(A)** and dynorphin A **(B)** were observed in the patient group. The difference for Leu-enkephalin was not significant **(C)**. Data were statistically analyzed by multi-way analysis of variance (ANOVA) with least significant difference (LSD) *post hoc* testing. Significant differences are indicated by asterisks: **p < 0.01, ***p < 0.001.

**Figure 2:**
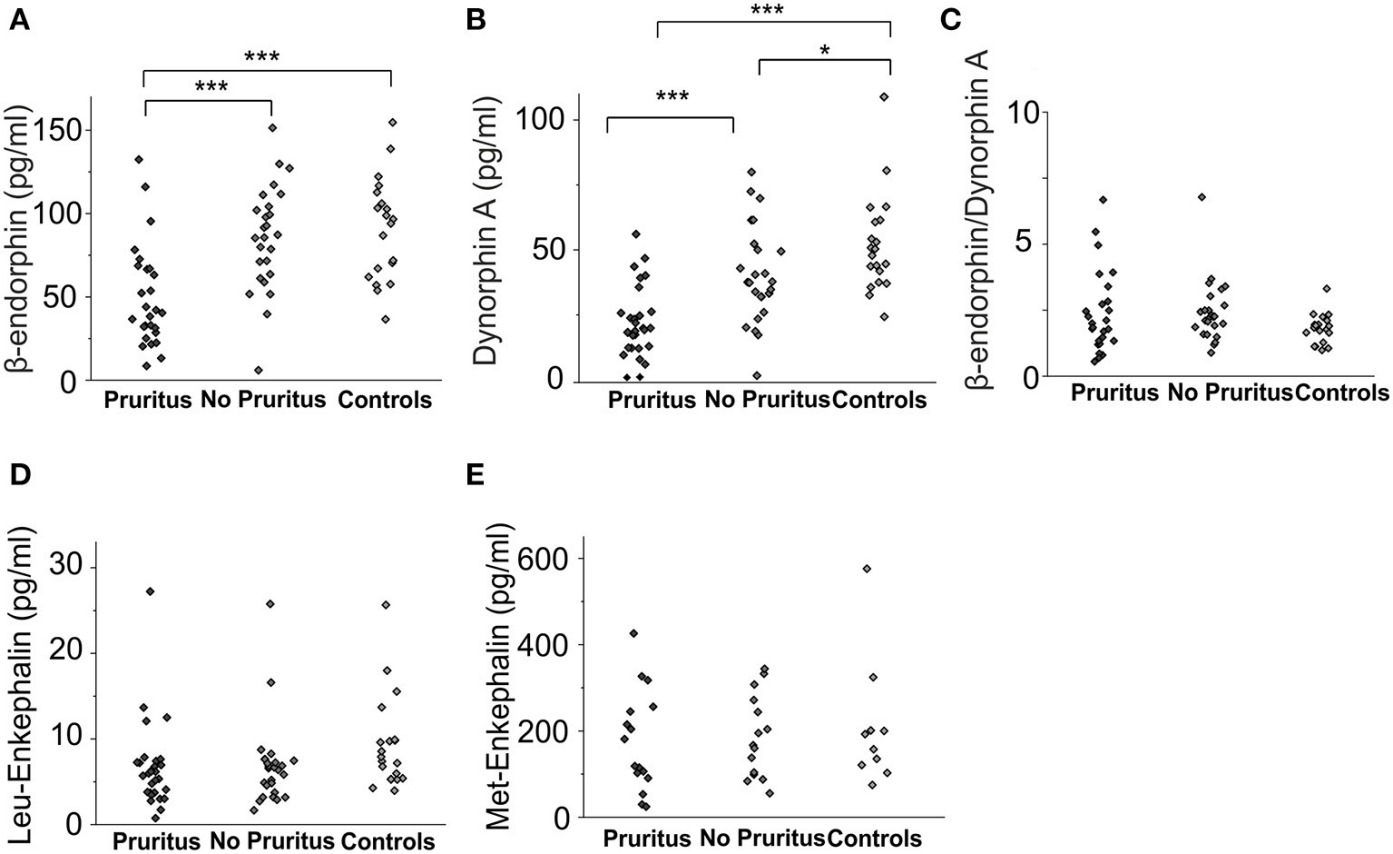
Endogenous opioid plasma levels in patients with cholestatic liver diseases with and without associated pruritus and healthy controls. Concentrations [pg/ml] of β-endorphin **(A)** and dynorphin A **(B)** in pruritic patients (*n* = 29), non-pruritic patients (*n* = 27) and healthy controls (*n* = 20) are presented in the respective subfigures. **(C)** presents the ratio of β-endorphin to dynorphin A levels for all groups **(C)**. Met-enkephalin and Leu-enkephalin levels in patients with and without pruritus as well as healthy controls are shown in subfigures **(D)** and **(E)**. Data were statistically analyzed by multi-way analysis of variance (ANOVA) with least significant difference (LSD) *post hoc* testing. Significant differences are indicated by asterisks: *p < 0.05, ***p < 0.001.

Concentrations of β-endorphin (67.3 ± 4.9 pg/ml vs. 90.5 ± 6.9 pg/ml, *p* < 0.01, ANOVA) and dynorphin A (30.3 ± 2.5 pg/ml vs. 52.1 ± 4.3 pg/ml, *p* < 0.001, ANOVA) were significantly lower in patients with cholestatic liver diseases in general compared to healthy controls (Figures 1A,B). The difference in Leu-enkephalin values between patients and controls did not reach significance (6.7 ± 0.7 pg/ml vs. 9.3 ± 1.2 pg/mlL, *p* = 0.053, Figure 1C). Met-enkephalin levels did not differ between patients and healthy controls (Figure 1D). Patients suffering from pruritus had significantly lower levels of β-endorphin (49.5 ± 5.9 pg/ml, Figure 2A) and dynorphin A (21.8 ± 2.6 pg/ml, Figure 2B) compared to patients without pruritus and healthy subjects, whereas the MOR/KOR-ratio was unaltered (Figure 2C). No significant differences were found here for Leu- and Met-enkephalin concentrations between all groups (Figures 2D,E).

### Leu-Enkephalin and Dynorphin A Levels Are Elevated in Patients With Cirrhosis

**Figure 3:**
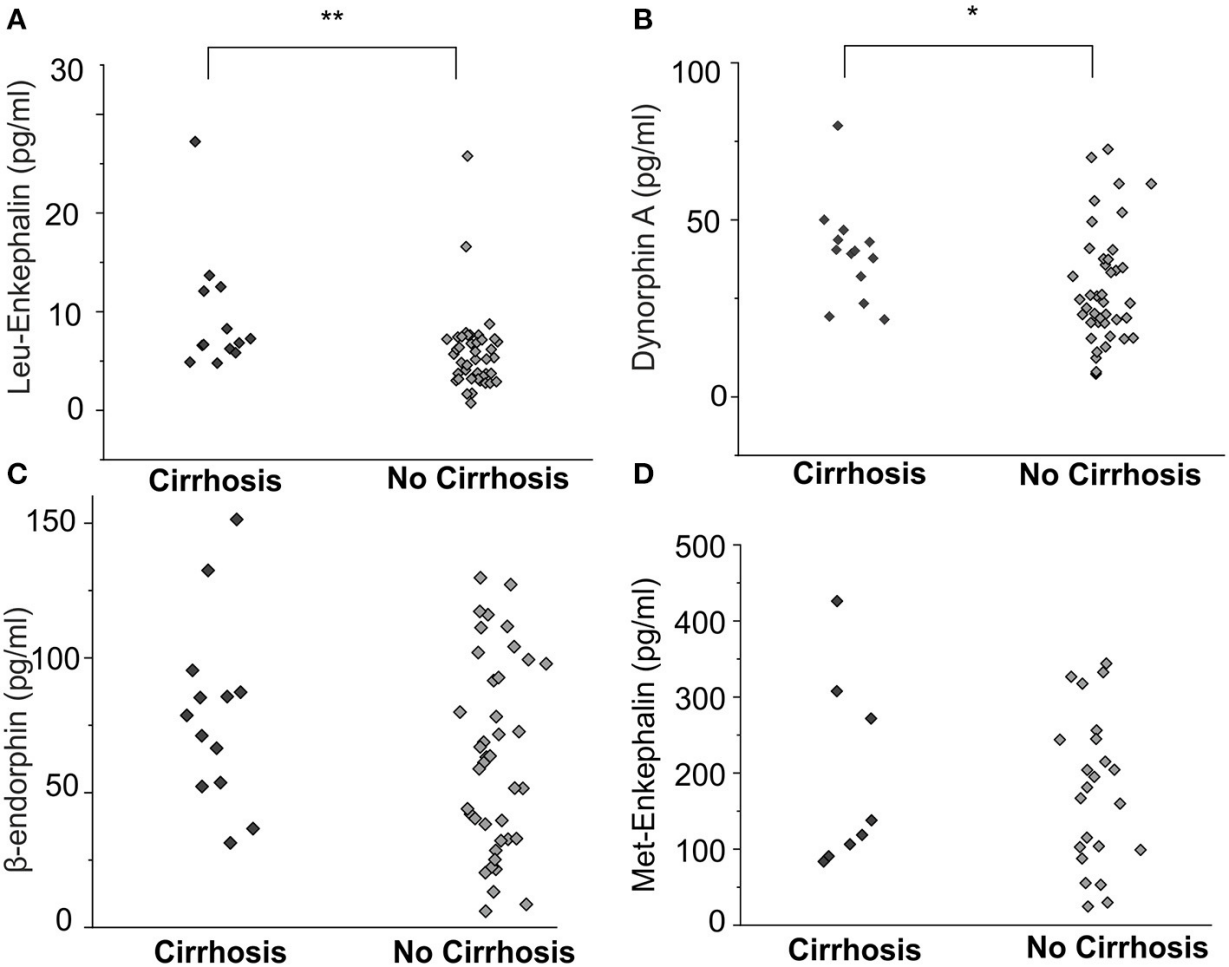
Endogenous opioid plasma levels in patients with cholestatic liver diseases with and without cirrhosis. Leu-enkephalin and dynorphin A concentrations [pg/ml] were significantly higher in patients with liver cirrhosis (**A, B**, *n* = 13) compared to non-cirrhotic patients (*n* = 43), but no significant differences were observed for β-endorphin and Met-enkephalin concentrations between those groups **(C,D)**. Data were statistically analyzed by multi-way analysis of variance (ANOVA) with least significant difference (LSD) *post hoc* testing. Significant differences are indicated by asterisks: *p < 0.05, **p < 0.01.

Cirrhotic patients (*n* = 13) had compared to non-cirrhotic patients (*n* = 43) significantly higher levels of MOR agonist Leu-enkephalin (10.6 ± 2.8 pg/ml vs. 5.8 ± 0.6 pg/ml, *p* < 0.01, ANOVA, Figure 3A) and KOR agonist dynorphin A (39.5 ± 4.6 ng/ml vs. 27.7 ± 2.9 ng/ml, *p* < 0.05, ANOVA, Figure 3B), but no significant differences were observed for β-endorphin and Met-enkephalin concentrations between those groups (Figures 3C,D).

### Endogenous Opioid Plasma Levels and Cholestasis Parameters do Not Correlate With Pruritus Intensity in Patients With Hepatic Pruritus

**Figure 4:**
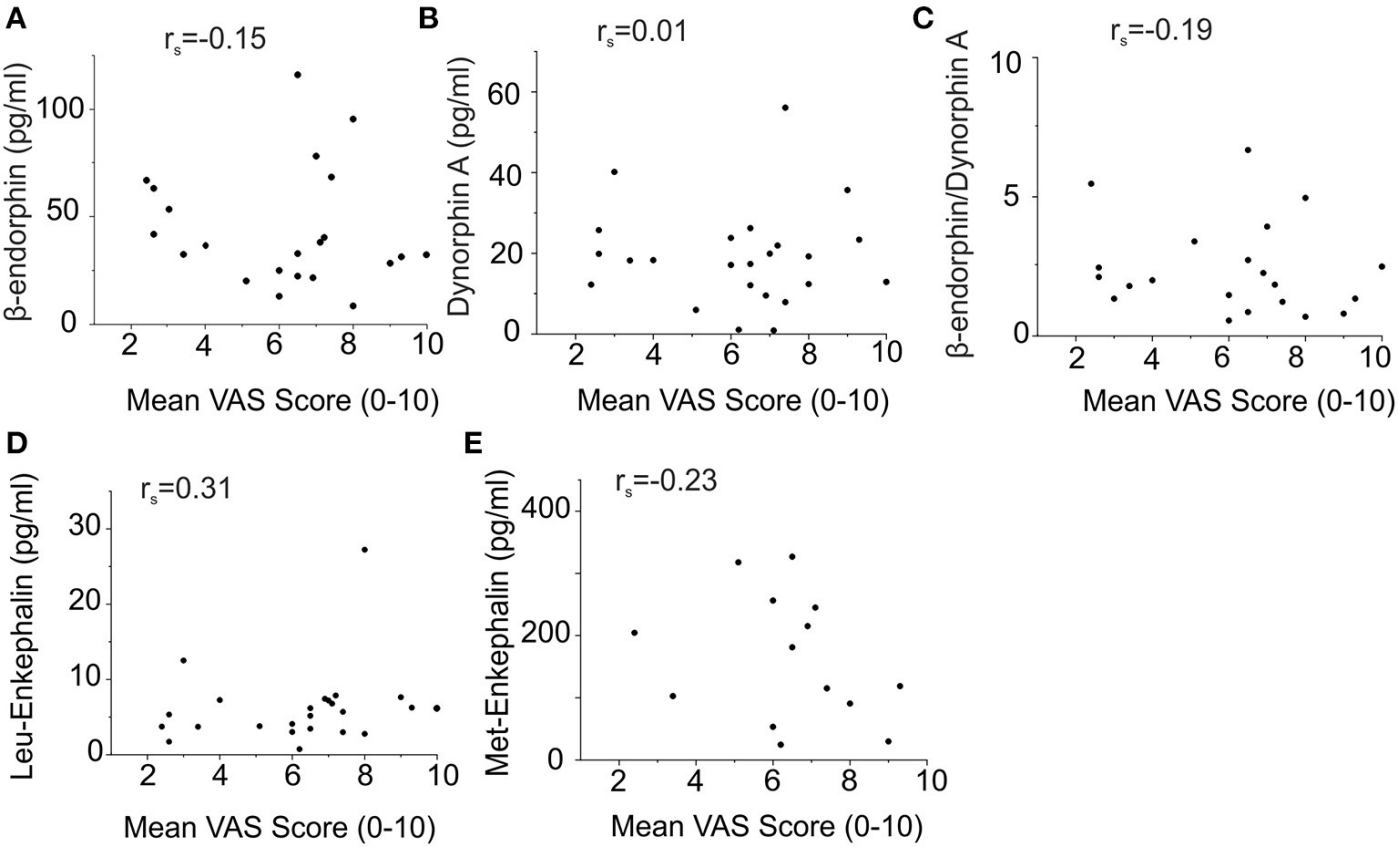
No correlation of endogenous opioid plasma levels with mean pruritus VAS scores of patients with hepatic pruritus. Symbols (circles) represent the opioid concentration and VAS score of the individual patients, respectively. Correlations were statistically calculated using Spearman's rank. Endogenous opioid levels did not correlate with the reported itch intensity assessed as the mean itch VAS score over the last week [**(A)** β-endorphin: r_s_ = −0.15; **(B)** dynorphin A: r_s_ = 0.01; **(C)** β-endorphin/dynorphin A ratio: r_s_ = −0.19; **(D)** Leu-enkephalin: r_s_ = 0.31; **(E)** Met-enkephalin: r_s_ = −0.23].

None of the analyzed endogenous opioids levels correlated with the reported itch intensity, assessed as the mean itch VAS score over the last week (Figure 4). We obtained comparable results when the numeric rating scale (NRS) score or Worst Itch (WI)-VAS score over the last week were chosen as itch intensity outcome measures.

We also did not observe correlations between the cholestasis markers γGT, AP, or total serum bilirubin and itch intensity (Supplementary Figure 1).

Levels of tested endogenous opioids were additionally evaluated for correlation with the parameters of γGT, AP, and total serum bilirubin. We could not detect a correlation between any cholestasis parameter and opioid subtype (Supplementary Figure 2).

### Effective Antipruritic Treatment Does Not Affect Endogenous Opioid Levels

**Figure 5:**
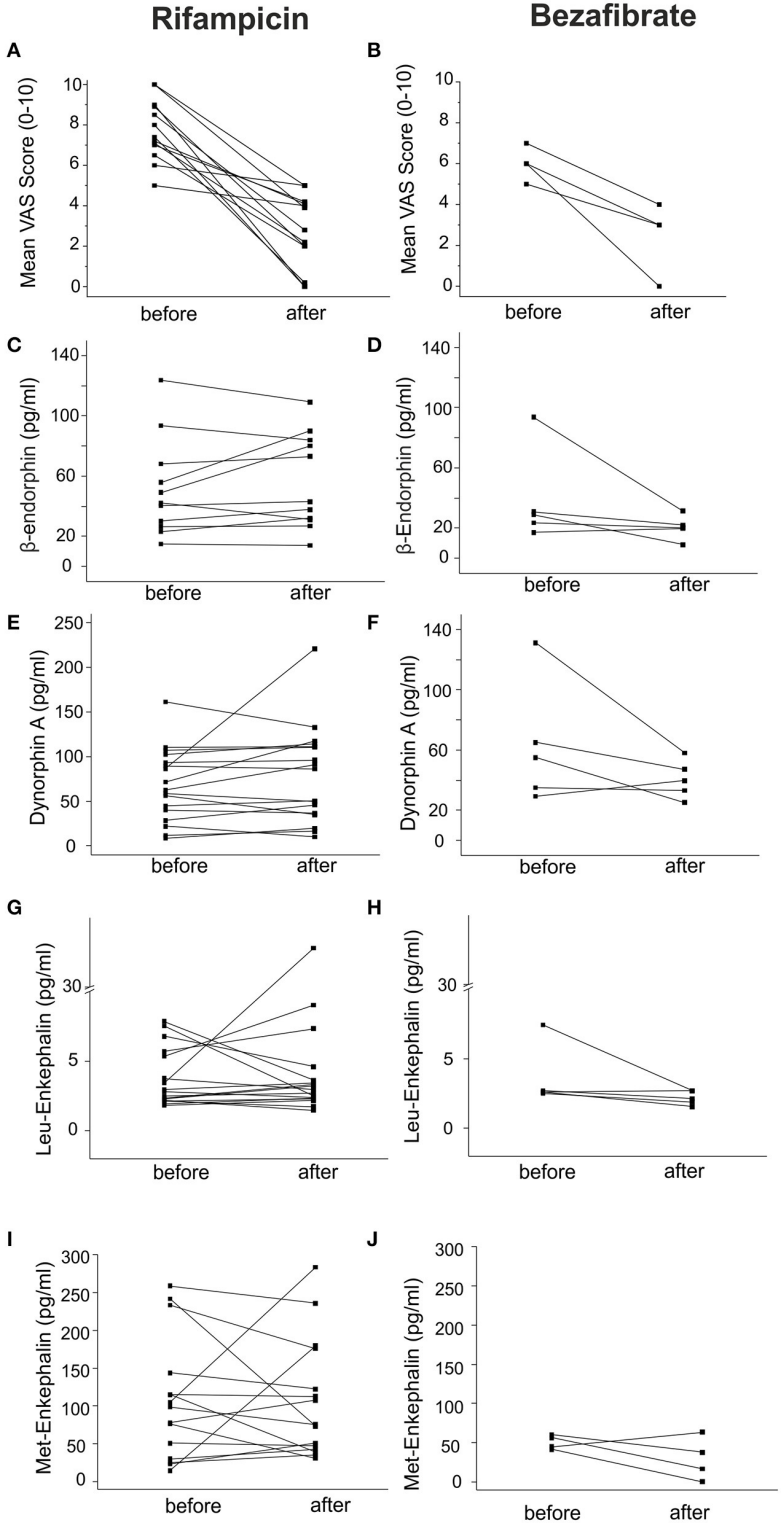
Endogenous opioid plasma levels in patients with cholestatic liver diseases before and after anti-pruritic treatment. Endogenous opioid levels were assessed in 22 patients treated with the anti-pruritic drugs rifampicin (150–300 mg qd) or bezafibrate (400 mg qd). Black squares represent individual patient values before and after treatment. **(A)** and **(B)** indicate the mean change of VAS over the last week before and after treatment with the respective drugs. Levels of β-endorphin, dynorphin A, Leu- and Met-enkephalin did not significantly change during treatment with both drugs **(C–J)**. Data were statistically analyzed by Wilcoxon test.

Twenty two patients were treated with rifampicin (150–300 mg qd) or bezafibrate (400 mg qd) for 2–6 weeks. We assessed the endogenous opioid levels before and after treatment. Itch intensity descreased on a VAS scale by 3.8 ± 0.9 and 3.5 ± 0.9 after treatment with rifampicin and bezafibrate, respectively (Figures 5A,B). Although patients experienced a clinically meaningful decrease in pruritus, opioid levels remained largely unaltered (Figures 5C–J and Table 3).

**Table 3:** Endogenous opioid concentrations in 22 patients before and after receiving anti-pruritic treatment with rifampicin (150–300 mg qd) or bezafibrate (400 mg qd).

**Opioid concentrations^a^**			***P*-value**
	**before rifampicin**	**after rifampicin**	
	***n*** **= 17**	***n*** **= 17**	
β-endorphin (pg/ml)	49.02 (38.3–68.2)	44.4 (32.2–80.8)	0.29
Dynorphin A (pg/ml)	62.5 (39.9–93.1)	86.1 (36.4–111.1)	0.65
Leu-enkephalin (pg/ml)	2.8 (2.2–5.4)	3.0 (2.3–3.7)	0.78
Met-enkephalin (pg/ml)	98.4 (40.4–129.5)	75.6 (45.3–148.8)	0.68
	**before bezafibrate**	**after bezafibrate**	
	***n*** **= 5**	***n*** **= 5**	
β-endorphin (pg/ml)	29.0 (23.5–30.9)	20.3 (19.8–22.0)	0.14
Dynorphin A (pg/ml)	54.9 (35.0–65.30)	39.6 (33.1–47.1)	0.08
Leu-enkephalin (pg/ml)	2.6 (2.6–2.7)	2.1 (1.9–2.7)	0.08
Met-enkephalin (pg/ml)	50.7 (44.1–57.4)	27.4 (12.9–44.2)	0.25

*Data were statistically analyzed by Wilcoxon-test*.

a *Data are shown as median (interquartile range)*.

## Discussion

In our study, dynorphin A and β-endorphin levels were both lower in pruritic patients with immune-mediated cholestatic liver diseases, whereas the MOR/KOR ligand ratio did not significantly differ from the control group.

Endogenous opioid peptides including endorphins, dynorphins and enkephalins are processed from three precursor molecules: proopiomelanocortin, prodynorphin, and proenkephalin, respectively (20). After proteolytic cleavage, they act upon binding different established opioid receptors. While dynorphins are generally described as endogenous agonists of κ-opioid receptors (KOR), endorphins and enkephalins show more affinity for μ-opioid receptors (MOR) (21).

The main location of endogenous opioid peptides and receptors is of course the central nervous system, but they are present as an established part of the enteric nervous system and other cell types (22). mRNA encoding the three endogenous opioid precursor molecules was found in several peripheral tissues, including the reproductive system, pancreas and immune cells (20). Expression and elevation of endogenous opioids in liver tissue and systemically in patients with cholestatic liver disease is limited to Met-enkephalin and enkephalin precursor molecules (23).

Opioid peptides are degraded enzymatically by various peptidases such as aminopeptidases, serine peptidases and angiotensin-converting enzyme (ACE) (24), of which some are present at the blood-brain-barrier (BBB). β-endorphin, Leu-enkephalin and Met-enkephalin cross the BBB via carrier systems such as P-glycoprotein (25, 26), which has not been reported for dynorphin A so far. It is conceivable that cholestasis alters expression or enzymatic activity of these peptidases resulting in reduced systemic levels of opioids.

There is convincing evidence for a role of the endogenous opioid system in itch transmission, for example, on the spinal level, with the μ-opioid system involved in pro-pruritic and the κ-opioid system in anti-pruritic pathways (27). It is well-known that especially epidural and spinal application of medical opioids induces itch as a side effect (8). Recent data suggest that cross-activation of the human spinal cord μ-opioid receptor 1Y isoform and Gastrin-releasing Peptide Receptor (GRPR) might contribute to opioid-induced itching (28). It was also shown that different stimuli, including painful stimuli and menthol, can activate BHLHB5+ inhibitory interneurons in the spinal cord with dynorphin representing one of their released neurotransmitters. Dynorphin might subsequently inhibit GRPR+ or other downstream neurons in the itch pathway (27, 29).

For hepatic pruritus, data on the pathophysiological involvement of endogenous opioids is limited and includes findings of elevated plasma opioid levels in rats with surgically induced cholestasis (10) and Met-enkephalin immunoreactivity in liver tissue of a few patients with PBC and chronic hepatitis C virus infection (30). Other reports, however, indicated an inverse correlation with higher endogenous opioid levels at advanced histological stages of PBC, when pruritus may be alleviated despite worsening of cholestasis (18, 31).

Drugs influencing the opioid system are applied for the treatment of pruritus in systemic diseases such as chronic kidney disease-associated pruritus (CKDaP) and hepatic pruritus (1). The oral μ-opioid antagonist naltrexone induced a mild antipruritic effect in two smaller randomized placebo-controlled trials (32, 33) in cholestatic patients. A meta-analysis, comparing the itch-reducing effect of several drugs, clearly indicated inferiority of naloxone and naltrexone compared to rifampicin (17). Nalfurafine, a κ-opioid agonist, is licensed in Japan, but not in Europe or USA, for treatment of CKDaP and hepatic pruritus (1). In a randomized, placebo-controlled trial including a rather inhomogeneous group of 318 patients with different liver diseases, treatment with nalfurafine resulted in a statistically significant but clinically questionable reduction of pruritus (16). This trial and other studies on hepatic pruritus (19, 34) included patients suffering from very diverse underlying liver diseases, ranging from chronic viral hepatitis, immune-mediated cholestatic disorders including overlap syndromes, to liver cirrhosis of various causes. The selection and examination of these inhomogeneous patient collectives might have resulted in divergent laboratory findings and reactions to treatment, which we hoped to minimize here by choosing well-defined patient and control groups.

Recently, the KALM-study showed a clear benefit using intravenously applied difelikefalin, a peripherally acting κ-opioid agonist, in CKDaP (35). A current phase II trial is investigating the effects of an oral galenic of difelikefalin on pruritus in PBC patients. While influencing peripheral opioid receptors represents an interesting mechanism, especially in CKDaP, clinical data have to be awaited on its efficiency in pruritus in liver disease.

Rifampicin is a potent anti-pruritic drug in hepatic pruritus (17, 36), although its mode of action, potentially via a pregnane X receptor (PXR)-dependent mechanism, remains to be resolved. Bezafibrate, an agonist of peroxisome proliferator-activated receptors (PPAR), used to treat dyslipidemia, was shown to also exert anti-inflammatory and anti-cholestatic properties and to improve pruritus in PBC patients (37). While both drugs convincingly reduced mean and individual itch intensity in pruritic patients in our study, we could clearly show that this effective treatment did not affect systemic endogenous opioid levels.

Thornten and colleagues reported on elevelated concentrations of Leu-enkephalin in plasma of patients with liver cirrhosis due to several causes (38). Our data supports this finding with mildly increased levels of Leu-enkephalin in cirrhotic patients. However, this is likely attributed to impaired liver function rather than involvement in hepatic itch transmission as we observed no correlation between itch intensity and Leu-enkephalin concentrations in our cohort.

Our study is limited to analyses of endogenous opioid levels in plasma of patients with chronic liver diseases. It raises the question for the underlying cause of reduced levels of β-endorphin and dynorphin A in pruritic patients.

As we observed lower levels in patients with liver diseases in general compared to healthy controls, opioid concentrations might be influenced by the pathological cholestatic conditions. The lower concentrations of KOR and MOR agonists in cholestatic patients may result from increased hepatic metabolism or increased renal clearance, which warrants further investigations in animal models of intrahepatic cholestasis. Additionally, we can neither rule out that the blood brain barrier is significantly altered during cholestasis nor that a central upregulation in opioid signaling might be present in patients with chronic pruritus, which could result in decreased peripheral endogenous opioid concentrations in terms of a negative feedback loop. While other data suggest increased production and distribution of endogenous opioids in liver (39) and skin (40) in cholestatic conditions, it might be helpful to further investigate skin or liver biopsies, for example, for opioid immune reactivity and opioid receptor expression. These samples are unfortunately not available for this set of patients. With a similar plasma MOR/KOR ligand ratio in pruritic and control groups in this study, an imbalance between pro- and anti-pruritogenic subtypes of endogenous opioids is unlikely to substantially contribute to the pathophysiology of hepatic pruritus. Although an imbalance on the level of the central nervous system cannot be excluded, our data do not support a major role of peripheral endogenous opioids in the pathogenesis of hepatic pruritus.

## Data Availability Statement

The original contributions presented in the study are included in the article/Supplementary Material, further inquiries can be directed to the corresponding author/s.

## Ethics Statement

The studies involving human participants were reviewed and approved by Ethikkommission Friedrich-Alexander-Universität Erlangen-Nürnberg. The patients/participants provided their written informed consent to participate in this study.

## Author Contributions

AK conceived the study. MD, KW, MV, PD, and AK collected blood specimen, clinical and laboratory patient data. MD performed ELISA analyses and wrote the manuscript. MN, AK, and PD supervised data analyses and preparation of the manuscript. All authors discussed and critically revised the manuscript.

## Conflict of Interest

The authors declare that the research was conducted in the absence of any commercial or financial relationships that could be construed as a potential conflict of interest.

## Supplementary Material

Supplementary Figure 1 No correlation of cholestatic parameters with itch intensity of patients with cholestatic liver diseases. The cholestatic parameters γGT **(A)**, AP **(B)**, and total serum bilirubin **(C)** did not correlate with the reported itch intensity assessed as the mean itch VAS score over the last week. Correlations were statistically calculated using Spearman's rank.

Supplementary Figure 2 No correlation of cholestatic parameters with endogenous opioid levels in patients with cholestatic liver diseases. Levels of all tested endogenous opioids were correlated with laboratory cholestasis markers γGT, AP, and total serum bilirubin of all included patients with cholestatic liver diseases (n = 56). No correlation between any opioid concentration and and cholestasis parameter was observed. Correlations were statistically calculated using Spearman's rank.
