# Supplementary figures and images for: Endogenous Opioid Levels Do Not Correlate With Itch Intensity and Therapeutic Interventions in Hepatic Pruritus
Source: Front Med (Lausanne). 2021 Apr 14;8:641163. doi: 10.3389/fmed.2021.641163 (PMC8079640; doi:10.3389/fmed.2021.641163)

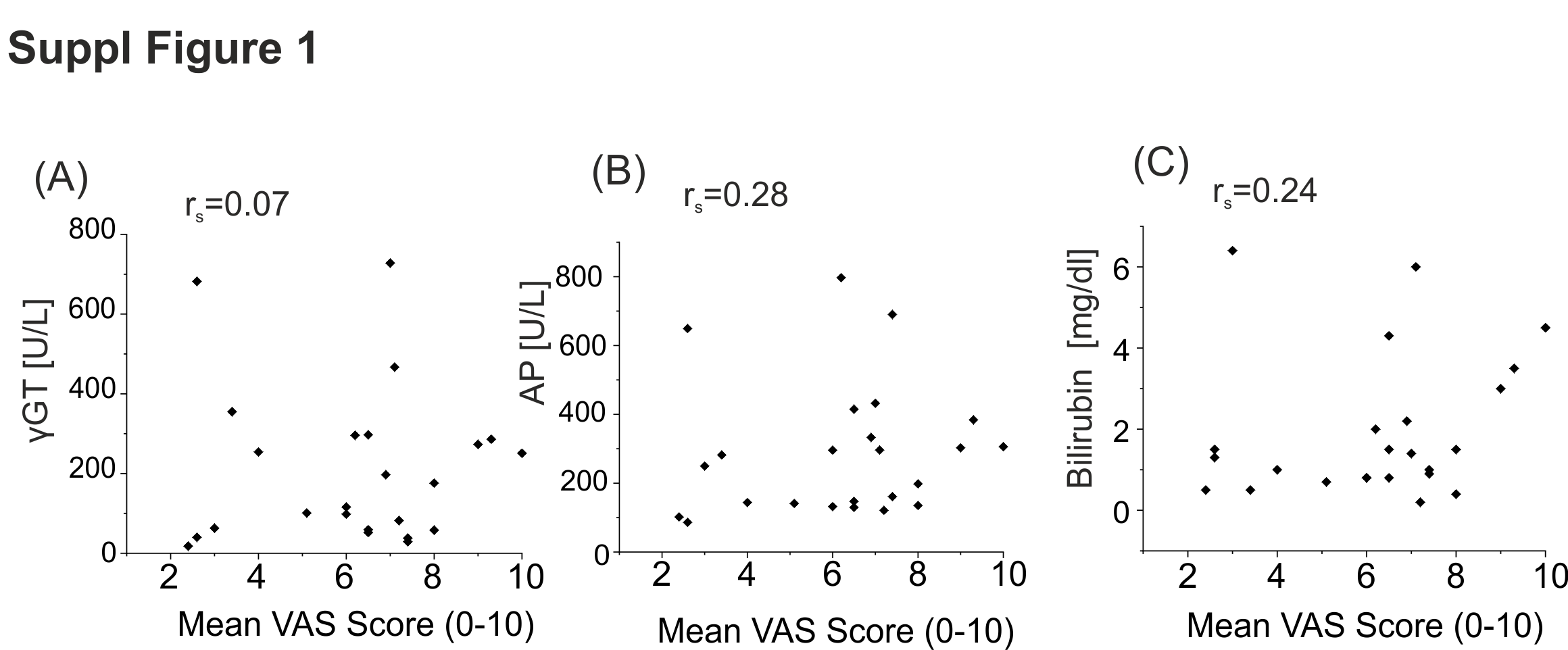

Supplement: Supplementary Figure 1 — No correlation of cholestatic parameters with itch intensity of patients with cholestatic liver diseases. The cholestatic parameters γGT (A), AP (B), and total serum bilirubin (C) did not correlate with the reported itch intensity assessed as the mean itch VAS score over the last week. Correlations were statistically calculated using Spearman's rank. [file Image_1.TIF]

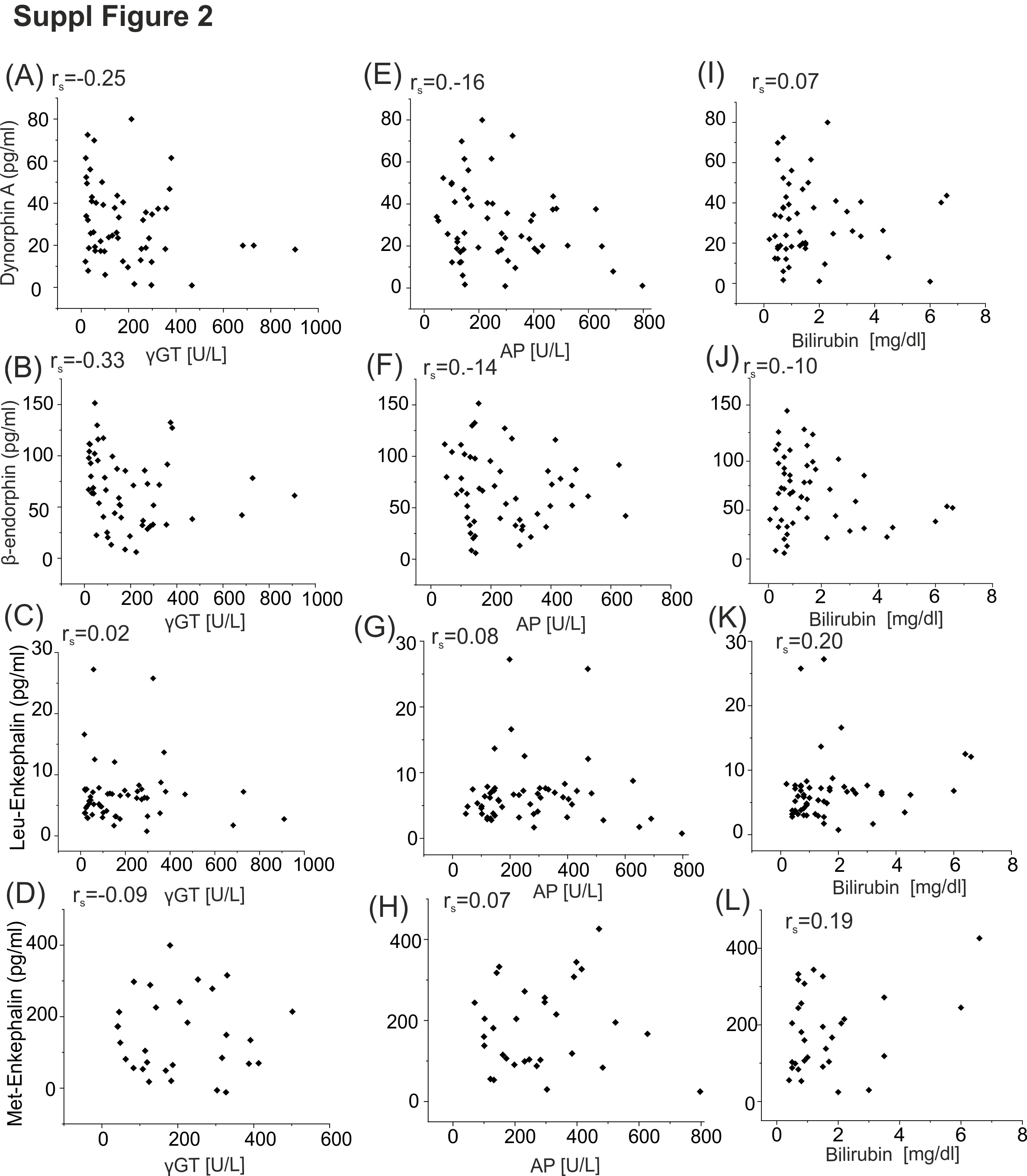

Supplement: Supplementary Figure 2 — No correlation of cholestatic parameters with endogenous opioid levels in patients with cholestatic liver diseases. Levels of all tested endogenous opioids were correlated with laboratory cholestasis markers γGT, AP, and total serum bilirubin of all included patients with cholestatic liver diseases (n = 56). No correlation between any opioid concentration and and cholestasis parameter was observed. Correlations were statistically calculated using Spearman's rank. [file Image_2.TIF]
